# Airborne SARS-CoV-2 RNA detected during deliveries with unmasked patients

**DOI:** 10.1016/j.infpip.2024.100389

**Published:** 2024-08-17

**Authors:** Sara Thuresson, Malin Alsved, Åsa Leijonhufvud, Andreas Herbst, Patrik Medstrand, Jakob Löndahl, Carl-Johan Fraenkel

**Affiliations:** a Division of Ergonomics and Aerosol Technology, Department of Design Sciences, Lund University, Lund, Sweden; b Department of Obstetrics and Gynecology Helsingborg Hospital, Lund University, Helsingborg, Sweden; c Department of Clinical Science Helsingborg, Lund University, Helsingborg, Sweden; d Department of Obstetrics and Gynecology, Skåne University Hospital, Lund University, Lund, Sweden; e Institution for Clinical Sciences Lund, Lund University, Lund, Sweden; f Department of Translational Medicine, Clinical Virology, Lund University, Lund, Sweden; g Department of Infection Control, Region Skåne, Lund, Sweden; h Division of Infection Medicine, Department of Clinical Sciences, Lund University, Lund, Sweden

**Keywords:** SARS-CoV-2, Aerosols, Childbirth, COVID-19

## Abstract

**Background:**

Healthcare workers in obstetric clinics may be exposed to airborne SARS-CoV-2 when treating patients with COVID-19.

**Method:**

In this study, performed during the midst of the pandemic, air samples were collected in delivery rooms during childbirth and analysed for SARS-CoV-2 RNA content.

**Result:**

Six of 28 samples collected inside delivery rooms were positive for SARS-CoV-2, but none in anterooms or corridors. Five of the six positive samples were from the same occasion.

**Discussion:**

This indicates that some patients could be major sources of exhaled virus, although the individual variation is large, and it is thus difficult to predict the risk of infection.

## Introduction

Numerous studies have investigated the presence of SARS-CoV-2 in hospital air to assess the risk of infection via inhalation when interacting with COVID-19 patients [[1], [2], [3]], but only a handful of studies have measured SARS-CoV-2 in delivery rooms [[4], [5], [6], [7]]. During childbirth, respiratory emissions are increased due to heavy breathing, potentially carrying infectious virus [8]. Moreover, the use of face masks in these situations is limited due to the physical exertion of pushing during delivery.

To date, published studies on airborne SARS-CoV-2 during delivery only include small numbers of air samples from childbirth (<25, often <10, per study) [[4], [5], [6], [7]]. For vaginal delivery, the total number of measured occasions is around 15, whereof airborne SARS-CoV-2 was only detected during 3. However, on several occasions patients wore a mask, which reduces respiratory emissions, and often room ventilation was high (up to 15 air exchanges per hour).

The aim of the current study was to further explore the presence of airborne SARS-CoV-2 RNA during vaginal delivery by patients without face masks.

## Methods

The research team was notified by clinicians when a mother who had recently tested positive for COVID-19 was about to give birth. Air samples were then collected at two hospitals in Southern Sweden during Nov 2020 to Jan 2022, before, during and after delivery over the course of 1–6 hours. The air samplers were placed 0.5–2.5 m from the mother's head, at a height of about 1 m. Samples were also collected from adjacent anterooms and corridors. The delivery rooms were constructed with an outside air delivery rate of 60–120 litres/second (∼3–6 air changes per hour), and the possibility to use an additional mobile High-Efficiency Particulate Air (HEPA)–filtration unit. Ventilation measurements or any use of a HEPA-filtration unit was not recorded in this study.

Liquid cyclones (Coriolis μ, Bertin Instruments) were used for air collection, operating at 200 L min^−1^ for 10 min, with 15 mL of phosphate-buffered saline solution as collection liquid. The collection liquid was concentrated using Amicon Ultra-15 centrifugal filter units (50 kDa cutoff, Merck Millipore) and stored at -80 °C until analysis. SARS-CoV-2 RNA was detected by real time reverse transcription polymerase chain reaction (RT-qPCR) performed as in our previous work [9].

### Ethics statement

The study was approved by the National Ethical Review Board in Sweden (number 2020-01396).

## Results and discussion

In total, 43 air samples were collected during six childbirths, of which 28 were from delivery rooms, 9 from anterooms, 5 from corridors and 1 from a canteen in the ward. The number of collected and positive samples per occasion, as well as patient characteristics, are presented in Table I.

**Table I:** Patient characteristics and collected air samples for each sampling occasion

Individual	Time spentin DR^a^ beforefirst sampling(hrs)	Days sincelast positivetest	Days sincesymptomonset	Symptomsat delivery	Anaesthesia^c^	SARS-CoV-2 positive/collected air samples			
						Total collectedfrom DR^a^	Beforepushing	Duringpushing	Afterdelivery
1	8	0	12	Nasal congestion	NO, ED	**0/4**	0/2	0/1	0/1
2	7.5	3^b^	3	Nasal congestion	NO	**0/6**	0/0	0/0	0/6
3	4	3	-	Asymptomatic	NO, S	**5/5**	2/2	2/2	1/1
4	11.5	0	2	Sore throat	NO, ED	**1/6**	1/2	0/2	0/2
5	4.5	2	5	“Minor symptoms”	NO	**0/4**	0/1	0/2	0/1
6	7	0	0	Low grade fever	NO, ED	**0/3**	0/1	0/2	0/0

a DR = delivery room.

b Tested negative on the day of delivery.

c NO= nitrous oxide; ED=epidural; S=spinal.

All air samples were positive from inside the room of patient 3, who never showed any symptoms but was positive by PCR. The father, who was present in the room, was also positive by PCR. The sample collected from the anteroom to patient 3's delivery room was negative, indicating limited spread of virus particles through the entrance door. Patient 4 was positive by rapid antigen test and had a sore throat, but only one positive air sample (before delivery) was found. Patient 4 was likely close to symptom onset, increasing the probability of a high viral load and subsequent higher risk of emitting airborne virus [9]. However, patients 2 and 6 were also early on in the disease but provided only negative air samples. Face masks were not worn by the patients on any of the occasions.

All positive samples were found at a distance of 1–2 m from the patient. RNA concentrations were low (Ct values 37.7–40.5), similar to concentrations usually found in hospital environments where COVID-19 patients are present [1,2]. All samples from anterooms and corridors were negative. The risk of false positives we consider as low; all negative controls were negative by RT-qPCR.

Compared to previous studies of SARS-CoV-2 in air, both during childbirth and from other hospital environments, the proportion of positive air samples in our study was high (21% compared to around 10%) [1]. However, this is mainly attributed to patient 3 where all five samples were positive. A comparable study revealed a similar pattern, with around 20 % positive samples in total whereof the majority from a single patient [5]. In a study performed in the same area during the same time period, 17 patients of 88 (19%) showed at least one SARS-CoV-2 RNA positive sample in collected patient room air, as compared to 2 of 6 (33%) in this study [9]. The results indicate that there are patients who potentially spread a lot of virus during childbirth, similar to covid-care in general, but it is difficult to predict who those will be. Healthcare personnel often have prolonged and close contact with patients during childbirth in rooms that are not primarily designed for infection prevention. This increases the risk of exposure to airborne virus, which motivates the use of face masks and other prevention strategies against airborne pathogens. Enhanced ventilation is one additional measure that could be used [9], and the negative results of all anteroom and corridor samples indicate that ventilation measures prevented long-range transport of virus outside of the patient room in this study.

## Conflicts of interest

No conflict of interest is reported from any of the authors.

## Source of funding

AFA insurance (grant numbers 220077 and 220155), The Swedish research council FORMAS (grant numbers 2020-01490 and 2022-00323), the 10.13039/501100004359Swedish Research Council (grant number 2022-00311) and 10.13039/501100009252SciLifeLab Pandemic Laboratory Preparedness (PLP) Program.

## Credit author statement

**Sara Thuresson:** Investigation, Writing-Original Draft, Writing-Review & Editing.

**Malin Alsved:** Investigation, Writing-Review & Editing.

**Åsa Leijonhufvud:** Conceptualization, Investigation, Writing-Review & Editing.

**Andreas Herbst:** Investigation, Writing-Review & Editing.

**Patrik Medstrand:** Investigation, Writing-Review & Editing, Funding acquisition.

**Jakob Löndahl:** Conceptualization, Investigation, Writing-Review & Editing, Funding acquisition.

**Carl-Johan Fraenkel:** Conceptualization, Investigation, Writing-Review & Editing.
